# The Effect of Lipopolysaccharide-Induced Experimental Bovine Mastitis on Clinical Parameters, Inflammatory Markers, and the Metabolome: A Kinetic Approach

**DOI:** 10.3389/fimmu.2018.01487

**Published:** 2018-06-25

**Authors:** Carl-Fredrik Johnzon, Josef Dahlberg, Ann-Marie Gustafson, Ida Waern, Ali A. Moazzami, Karin Östensson, Gunnar Pejler

**Affiliations:** ^1^Department of Anatomy, Physiology and Biochemistry, Swedish University of Agricultural Sciences, Uppsala, Sweden; ^2^Department of Animal Nutrition and Management, Swedish University of Agricultural Sciences, Uppsala, Sweden; ^3^Department of Medical Biochemistry and Microbiology, Uppsala University, Uppsala, Sweden; ^4^Department of Molecular Sciences, Swedish University of Agricultural Sciences, Uppsala, Sweden; ^5^Department of Clinical Sciences, Swedish University of Agricultural Sciences, Uppsala, Sweden

**Keywords:** bovine mastitis, lipopolysaccharide, metabolomics, cytokines, short-chain fatty acids

## Abstract

Mastitis is an inflammatory condition of the mammary tissue and represents a major problem for the dairy industry worldwide. The present study was undertaken to study how experimentally induced acute bovine mastitis affects inflammatory parameters and changes in the metabolome. To this end, we induced experimental mastitis in nine cows by intramammary infusion of 100 µg purified *Escherichia coli* lipopolysaccharide (LPS) followed by kinetic assessments of cytokine responses (by enzyme-linked immunosorbent assay), changes in the metabolome (assessed by nuclear magnetic resonance), clinical parameters (heat, local pain perception, redness, swelling, rectal temperature, clot formation, and color changes in the milk), and milk somatic cell counts, at several time points post LPS infusion. Intramammary LPS infusion induced clinical signs of mastitis, which started from 2 h post infusion and had returned to normal levels within 24–72 h. Milk changes were seen with a delay compared with the clinical signs and persisted for a longer time. In parallel, induction of IL-6 and TNF-α were seen in milk, and there was also a transient elevation of plasma IL-6 whereas plasma TNF-α was not significantly elevated. In addition, a robust increase in CCL2 was seen in the milk of LPS-infused cows, whereas G-CSF, CXCL1, and histamine in milk were unaffected. By using a metabolomics approach, a transient increase of plasma lactose was seen in LPS-induced cows. In plasma, significant reductions in ketone bodies (3-hydroxybutyrate and acetoacetate) and decreased levels of short-chain fatty acids, known to be major products released from the gut microbiota, were observed after LPS infusion; a profound reduction of plasma citrate was also seen. Intramammary LPS infusion also caused major changes in the milk metabolome, although with a delay in comparison with plasma, including a reduction of lactose. We conclude that the LPS-induced acute mastitis rapidly affects the plasma metabolome and cytokine induction with similar kinetics as the development of the clinical signs, whereas the corresponding effects in milk occurred with a delay.

## Introduction

Mastitis is defined as an inflammation of the mammary tissue. In clinical mastitis, the symptoms follow the characteristic signs of inflammation: swelling, redness, and heat in the udder, accompanied by pain, and changes in color and the appearance of clots and increases in cell counts in the milk (1–4). In cows, mastitis is commonly the response to an intramammary infection (IMI) (5).

Mastitis is common worldwide, though the prevalence and incidence rates vary greatly (6–11). Mastitis is the most costly disease in the dairy industry. It causes losses through reduced milk yield and quality as well as cost of drugs, veterinary services, diagnostics, and culling of incurable cases (12). Furthermore, the use of antibiotics to treat mastitis is widespread (13, 14).

Mastitis is diagnosed by analyzing the milk for elevated somatic cell counts (SCCs), typically in combination with bacteriological examination by either phenotypical (milk cultures) or genotypical (PCR) methods (3, 15). Numerous previous studies have investigated the immune mediators involved in the inflammatory response to an IMI. In these studies, cytokines, acute phase proteins [serum amyloid A, haptoglobin, and lipopolysaccharide (LPS)-binding protein], and eicosanoids have been detected in milk from mastitic udders [comprehensively reviewed in Ref. (16)].

There is currently a growing interest in how the metabolome is affected by different pathological conditions (17). In bovine mastitis, there are several reports describing how the milk metabolome is affected by mastitis caused by live bacterial pathogens (18–21). However, although the blood metabolome of healthy animals has been characterized [reviewed in Ref. (22)], there is to our knowledge currently no information of how the plasma metabolome is affected during mastitis.

Although previous studies have identified several inflammation markers appearing in the milk of cows afflicted by mastitis (23, 24), there is more limited information on how the mastitis affects systemic inflammatory parameters. Moreover, there is limited insight into the kinetics of the inflammatory response during mastitis and how the changes in the levels of inflammatory markers and metabolites correlate with clinical signs. To address these issues, changes in the metabolite and immune mediator profiles were studied in cows challenged with LPS in the udder. Intramammary LPS infusion represents an established *in vivo* model for bovine mastitis. This model system has been used to study mastitis in a wide range of contexts (25–33).

## Materials and Methods

### Selection Criteria and Ethical Permit

Enrollment for the purposes of this study was limited to primiparous lactating cows. The animals were selected for the study based on milk SCCs < 100,000 cells/ml during the three monthly recordings immediately preceding the study. All animals were clinically healthy and showed no signs of mastitis. All procedures involving animals were performed at the Swedish Livestock Research Centre (Uppsala-Lövsta, Sweden) and were approved by the local ethical committee (ethical permit C22/15).

### Animals and Management

Two individual experiments, with identical set up were performed. In the first experiment, eight cows were included. In the second experiment, 10 cows were included. Each experiment included equal numbers of Swedish Red Breed and Swedish Holstein (SLB). In experiment I, individuals were (mean ± SD) 33.75 ± 2.65 months old and 185.8 ± 55.10 days into lactation. The quarters selected for LPS infusion had an average SCC of 44,500 ± 20,000 cells/ml based on the measurements taken during the three sampling points prior to infusion. In experiment II, individuals were 30.9 ± 1.8 months old and 167.3 ± 45.2 days into lactation. The quarters selected for LPS infusion had an average SCC of 26,000 ± 11,230 cells/ml based on the measurements taken during the three sampling points prior to infusion.

The cows were housed in a loose housing system, had *ad libitum* access to roughage and minerals, and were milked automatically. In experiment I, the cows were milked by a voluntary milking system (VMS) (DeLaval VMS, DeLaval AB, Tumba, Sweden). Cows in experiment II were milked by an automatic milking rotary system (DeLaval AMR, DeLaval AB, Tumba, Sweden) with batch milking twice a day at predetermined time points (05:00 and 16:00). The herd is regularly grouped according to the two different milking systems, but the housing conditions are identical for all cows. The VMS and AMR were located adjacent to each other inside the same temperature-controlled barn building.

One day prior to LPS infusion, the animals were moved to a tie-stall where they were tethered inside cubicles. This enables easier sampling, ensures greater behavioral uniformity, and allows easier health monitoring. On the day of infusion and the following day, the cows were machine milked twice a day in the tie-stall by the staff at the Livestock Research Centre. After the 24 h sampling point, the cows were returned to the loose housing system stall once they had recovered. The tie-stall is located in the same building as the loose housing system.

### Study Design and Intramammary Infusion

The cows were randomly allocated to one of the two infusion treatment groups (LPS and Control). Cows in the LPS treatment groups received 100 µg purified LPS (*Escherichia coli* serotype O111:B4; L3024 Sigma-Aldrich) dissolved in 10 ml physiological saline solution (0.9% NaCl for parenteral use; Braun) intramammary. Cows in the control group received an equivalent volume of sterile physiological saline solution. The LPS solution was prepared approximately 2 h prior to infusion. All solutions were brought to room temperature before use.

**Figure 1:**
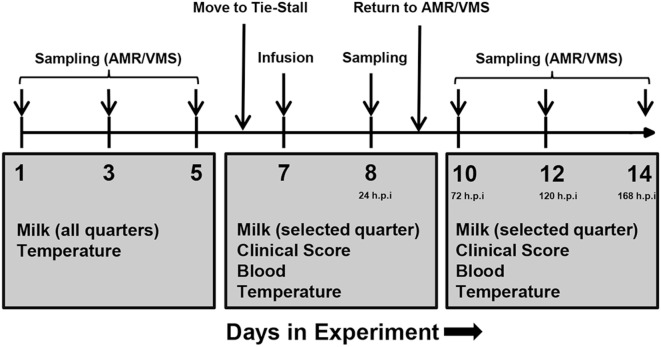
Experimental plan of infusion and sampling. One week prior to infusion, milk was collected from all udder quarters, and somatic cell counts were determined in order to assess health status. One healthy quarter per animal was selected for infusion. On day 6, cows were transferred from the loose housing system to a tie-stall. On day 7, lipopolysaccharide or physiological saline solution was infused and sampling was performed as indicated. Follow-up sampling was performed for 1 week after infusion. Abbreviations: AMR, automatic milking rotary; VMS, Voluntary Milking System; h.p.i, hours post-infusion.

Each study was performed over a period of 2 weeks (Figure 1). One week prior to infusion (days 1, 3, and 5), milk samples were taken from all quarters and screened for milk SCCs. The quarter with the lowest SCCs on each animal was selected for infusion. Infusions were performed on day 7. The selected udder quarter was prepared for intramammary infusion by cleaning the teat with a disposable udder towel and sterilization of the teat tip with 70% ethanol. Thereafter, the LPS or saline was infused through the teat canal using a TomCat catheter (31/2 Fr, 1.16 mm; Kendall, Tyco Healthcare, USA). Following infusion, the solution was distributed throughout the tissue by massaging the quarter.

Samples were taken immediately prior to infusion (“0 h”), 1, 2, 4, and 24 h post-infusion and additionally at days 10, 12, and 14 of the experiment (corresponding to 72, 120, and 168 h post-infusion). Milk was only taken from the infused quarter.

### Sampling and Clinical Examination

Sampling was consistently performed in the following order: milk, clinical examination, blood, and temperature. Prior to milk sampling, the teat was cleaned with an udder towel (DeLaval) followed by sterilization of the teat tip with 70% ethanol. Milk samples were collected in Falcon tubes (15 ml; Sarstedt). The progress of the inflammatory reaction was assessed by clinical scoring (Table S1 in Supplementary Material). The udder was examined by observation and palpation and the clinical signs were judged in terms of heat, pain, redness, and swelling. To determine changes in the milk, a small volume was deposited into a black test vessel, and the changes were judged visually. Blood samples were taken from the tail vein using vacuum tubes (7 ml Vacutainer Li-Heparin; BD) and vacuum cannula (Vacutainer 0.9 mm × 38 mm; BD). Temperature was monitored rectally using a digital thermometer.

For milk cell count analysis, 10 ml of whole milk was mixed with 30 µl 10% bronopol solution (Sigma-Aldrich) and stored at 4°C until sent for counting (maximum storage period of 6 days). Skim milk was prepared from the remaining milk. To this end, the milk was first centrifuged at 2,000 × *g* for 15 min at 4°C. The skim milk was then harvested by taking the layer between the fat layer and the cell pellet using long thin glass Pasteur pipettes. Milk cell counts were performed at the Department of Animal Nutrition and Management (HUV) at the Swedish University of Agricultural Sciences using an automatic milk somatic cell counter (Fossomatic 5000; FOSS—in the first study; FTIR 300HP, Perten Instruments—in the second study). The blood was centrifuged at 1,500 *g* for 30 min at 4°C and the plasma was removed by pipetting.

All samples were aliquoted into sterile 2 ml SafeSeal microtubes (Sarstedt). Prior to processing, samples were kept on ice until one round of sampling had been completed, after which the samples were transferred to 4°C storage until processing (for a maximum period of 5–6 h). The processed samples were subsequently aliquoted and stored at −80°C.

### Enzyme-Linked Immunosorbent Assays (ELISAs)

Concentrations of cytokines (IL-6, TNF-α, CCL2, G-CSF, and CXCL1) and histamine were determined in milk and plasma using ELISAs. ELISAs for TNF-α and IL-6 (R&D Systems) were performed according to the manufacturers’ instructions with the following modifications: 96-well microtiter plates (Immunoplate MaxiSorp; Nunc) were coated with capture antibody for 16–17 h. Blocking was allowed to proceed for 2.5–3 h. For milk, phosphate-buffered saline (PBS) supplemented with 5% Tween-20 and 25% skim milk (IL-6) or PBS supplemented with 5% Tween-20 and 75% skim milk (TNF-α) were used as sample and standard diluent. For plasma, PBS supplemented with 5% Tween-20 was used as a sample and standard diluent. CCL2 ELISAs (VetSet; Kingfisher Biotech) were performed according to the manufacturers’ instructions with the following modifications: PBS supplemented with 4% bovine serum albumin (BSA) was used as a reagent diluent. G-CSF, CXCL1 (MyBioSource) and histamine ELISAs (AH Diagnostics; EIA-4616 for milk, EIA-4005 for plasma) were performed according to the manufacturers’ instructions. All buffers were prepared fresh. PBS was prepared from tablets (Medicago). Tween-20 (Merck) and BSA (Fraction V; Roche Diagnostics) for buffers were always taken from fresh stocks. Skim milk was prepared from commercially available low pasteurized unhomogenized whole milk as described above.

### Metabolomics

Metabolites were extracted from skim milk by addition of 2 ml of chloroform/methanol (1:3 v/v) to 500 µl of skim milk. Following thorough vortexing, the mixture was centrifuged at 2,500 *g* for 15 min and 1 ml of supernatant (water/methanol) was transferred to a new tube. The supernatant was dried using a centrifugal evaporator, and the pellet was reconstituted in 520 µl phosphate buffer (0.135 M; pH 7) followed by addition of 50 µl of D_2_O and 30 µl nuclear magnetic resonance (NMR) internal standard (5.8 mM). Each sample mixture was transferred to a 5 mm NMR tube.

The details for NMR-based metabolomics analysis of plasma samples have been previously described (34). In brief, plasma samples were prepared for metabolite analysis after removal of plasma proteins through ultrafiltration at 13,000 *g* for 30 min (Nanosep, 3 kDa cut-off, Pall Life Science, Port Washington, NY, USA). The filters were washed eight times with water (500 µl, 36°C, and 2,000 *g*) to remove residual glycerol prior to application. Sixty microliters of plasma was then filtered. Subsequently, 40 µl of plasma filtrate was mixed with 55 µl Millipore water, 50 µl sodium phosphate buffer (pH 7), 15 µl D_2_O, and 10 µl NMR internal standard (5.8 mM). Each sample mixture was transferred to a 3 mm NMR tube.

The ^1^H NMR spectrum of milk and plasma samples was acquired using a Bruker Avance III spectrometer operating at 600 MHz proton frequency equipped with cryogenically cooled probe. Spectra were recorded at 25°C with 128 transients for milk and 512 transients for plasma, 4 s relaxation, 65,536 data points, and spectral width of 17,942 Hz using a zgesgp pulse sequence. All NMR spectra were manually processed using NMR Suite Professional Software (ChenomX Inc., Edmonton, AB, Canada). Processing included baseline correction and phase correction. For milk samples, each spectrum was integrated into 0.01 ppm integral regions (buckets) between 0.50 and 8.75 ppm, in which areas between 4.50 and 5.30 ppm were excluded. Each spectral region was then normalized to the sum of the total intensities before multivariate statistical analysis. The identification of NMR signals for milk samples was performed using Human Metabolome Data Base. For plasma samples, 48 metabolites were identified and manually quantified as previously described (34).

### Multivariate Statistical Analysis of Metabolomics Data

Multivariate statistical analyses, i.e., principal component analysis (PCA) partial least-squares-discriminant analysis (PLS-DA) were performed for metabolomics data from milk and plasma samples using SIMCA 14.0 software (Umetrics, Umeå, Sweden) (35). Comparison was made separately for each time point between LPS and control. The presences of outliers were investigated using Hotelling T2 Ellipse (95% CI). The validity of PLS-DA was tested using R^2^Y and Q^2^ values of the model. Variable influences on projection (VIP > 1) values from PLS-DA model were used to determine the most important discriminative metabolites in each comparison. The spectral region in milk samples or metabolites concentrations in plasma samples, which were found different between LPS and control in multivariate analysis were further compared using univariate statistical analysis at all time-points as describe below.

### Univariate Statistical Analysis

Data are shown as means (±SD). The data from the two studies was pooled and analyzed by multiple *t*-tests using the Holm–Sidak method (interval/ratio) or Mann–Whitney (ordinal). Outliers were identified and removed using the ROUT method. Differences were considered significant if *p* < 0.05.

## Results

### The Kinetics of the Development of Clinical Signs of Acute Mastitis After Intramammary LPS Infusion

**Figure 2:**
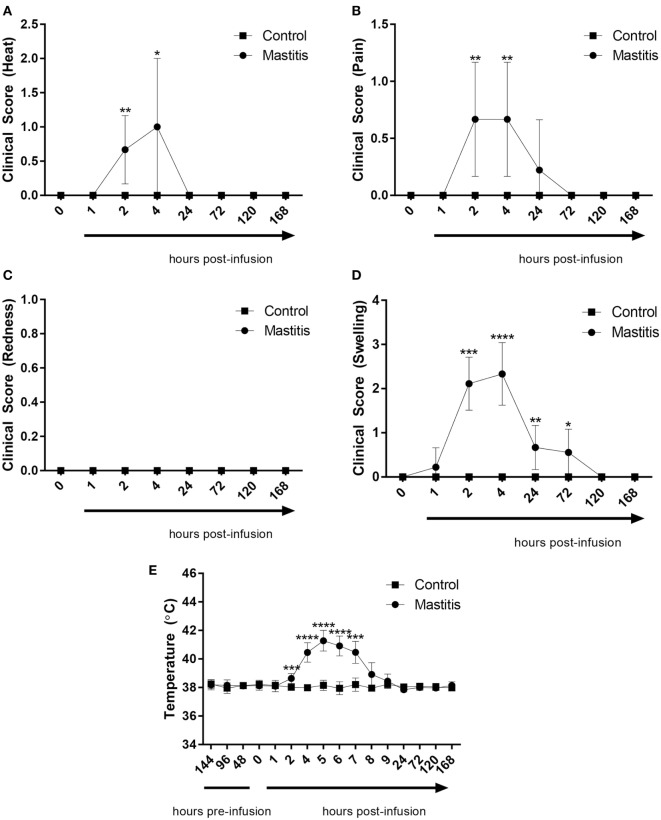
Clinical score and body temperature before and after infusion with lipopolysaccharide (LPS) or physiological saline solution. Immediately before and 1, 2, 4, 24, 72, 120, and 168 h post LPS infusion, the cows were scored on a scale of 0–3 for **(A)** heat (*n* = 7–9), **(B)** pain (*n* = 9), **(C)** redness (*n* = 9), and **(D)** swelling (*n* = 7–9). Temperature **(E)** was monitored rectally over the same period (*n* = 4–9). Results are given as mean (±SD). *p < 0.05; **p < 0.01; ***p < 0.001; p**** < 0.0001 [statistical analysis **(A–D)**: Mann–Whitney; **(E)**: multiple *t*-test].

To induce experimental mastitis, bacterial LPS was infused into one udder quarter per cow, followed by assessment of clinical signs of mastitis over a 7-day period. Within 2 h of intramammary LPS infusion, the temperature of the infused udder quarter perceptibly increased but returned to baseline levels within 24 h (Figure 2A; Table S1 in Supplementary Material). During this time period, the cows also showed signs of local pain perception, which persisted up to 24 h after LPS infusion (Figure 2B; Table S1 in Supplementary Material). Redness was not observed at any point during the experiment (Figure 2C; Table S1 in Supplementary Material). Quarters infused with LPS also showed signs of swelling. Swelling was observed starting 1 h after LPS infusion with a maximum at 2–4 h, and remained perceptible up to 72 h post-infusion (Figure 2D; Table S1 in Supplementary Material). The body temperature began to increase 2 h post-infusion, peaking at 5 h post-infusion with temperatures of 41.5°C recorded in some animals (Figure 2E). The body temperature returned to normal levels 24 h post-infusion in all animals infused with LPS (Figure 2E). Control animals (infused with saline solution) did not exhibit changes in clinical scoring or increased body temperature (Figures 2A–E).

**Figure 3:**
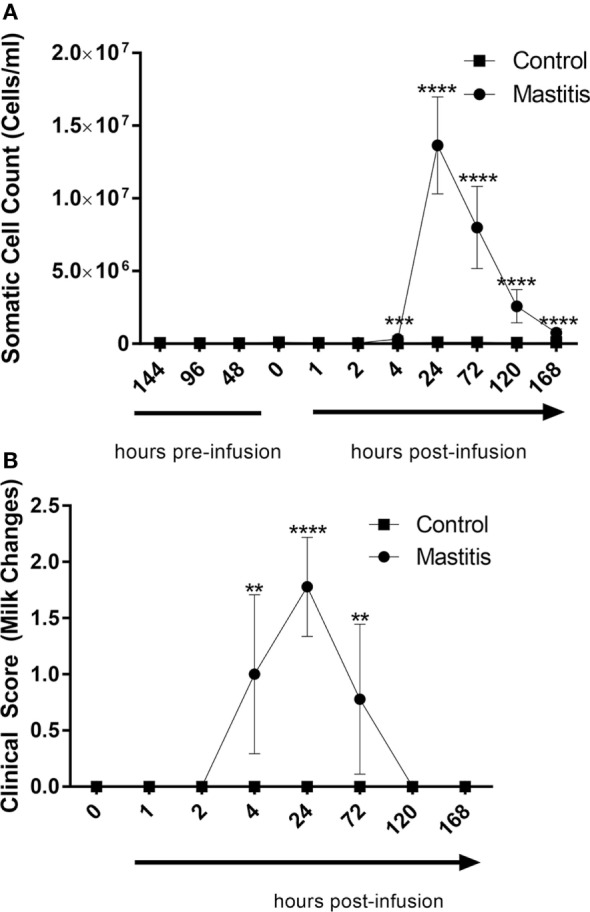
Milk somatic cell counts (SCCs) and milk change scores before and after infusion with lipopolysaccharide or physiological saline solution. **(A)** SCCs were determined using an automatic milk cell counter (*n* = 6–9). **(B)** Milk changes were scored on a scale of 0–3 (*n* = 9). Results are given as mean (±SD). *p < 0.05; **p < 0.01; ***p < 0. 001; ****p < 0.0001 [statistical analysis **(A)**: multiple *t*-test; **(B)**: Mann–Whitney].

Intramammary LPS infusion caused a dramatic rise of the milk SCC, increasing from levels < 100,000 cells/ml to 1.6 × 10^7^ cells/ml 24 h post-infusion (Figure 3A). Milk SCC remained elevated during 1 week after LPS infusion. Changes in milk color and the appearance of clots were first observed 4 h after LPS infusion, peaked at 24 h, and remained visible at 72 h after LPS infusion (Figure 3B). Milk samples from control non-infused animals did not exhibit increased SCC or any other changes (Figures 3A,B).

### Cytokine Concentrations in Milk and Plasma

**Figure 4:**
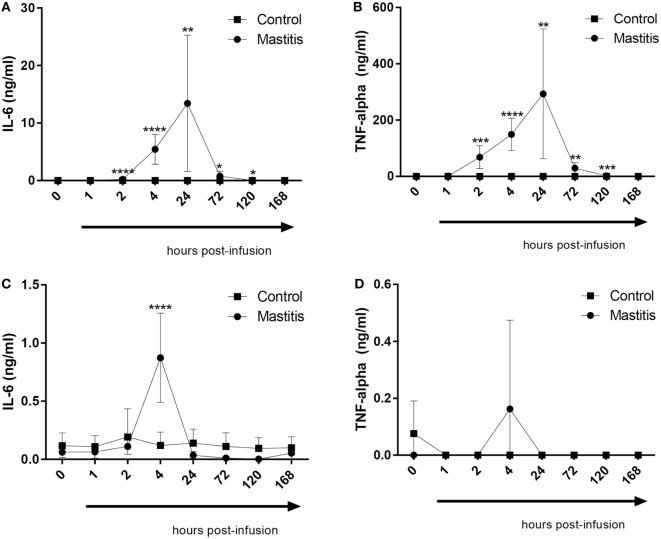
Concentrations of IL-6 and TNF-α measured by enzyme-linked immunosorbent assay in milk and plasma before and after infusion with lipopolysaccharide or physiological saline solution. **(A)** IL-6 concentrations measured in milk (*n* = 8–9). **(B)** TNF-α concentrations measured in milk (*n* = 7–9). **(C)** IL-6 concentrations measured in plasma (*n* = 6–9). **(D)** TNF-α concentrations measured in plasma (*n* = 7–9). Results are given as mean (±SD). *p < 0.05; **p < 0.01; ***p < 0.001; ****p < 0.0001 (statistical analysis: multiple *t*-test).

To study the output of cytokines induced by experimental mastitis, we first measured the levels in milk of IL-6 and TNF-α, both of which are well-established inflammatory markers. These analyses showed that the levels of IL-6 in milk were dramatically increased after LPS infusion, peaking between 4 and 24 h post infusion, after which, a gradual decline to baseline levels was seen (Figure 4A). LPS infusion also caused a dramatic increase in the levels of TNF-α in milk; the TNF-α response showed similar kinetics as seen for IL-6 (Figure 4B). In plasma, a significant elevation of IL-6 levels was seen at 4 h after LPS infusion, after which the IL-6 levels returned to baseline (Figure 4C). In contrast to IL-6, no significant increase in plasma TNF-α was seen after LPS infusion, although a trend of increased TNF-α was seen at 4 h post-infusion (Figure 4D).

**Figure 5:**
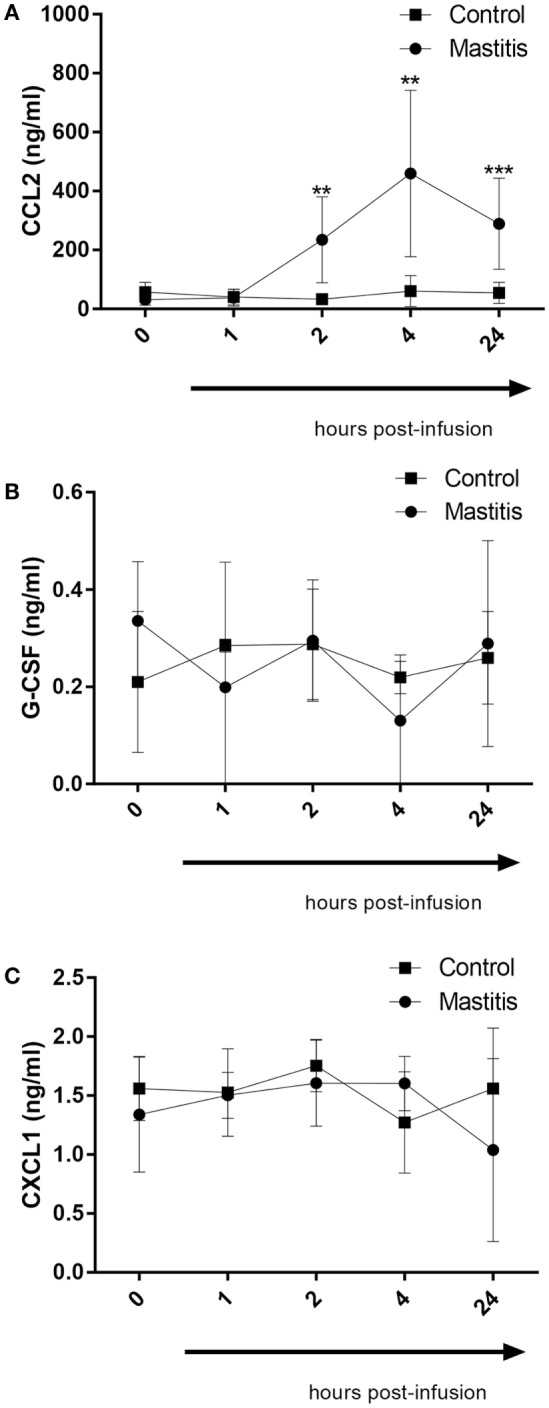
Concentrations of CCL2, G-CSF, and CXCL1 measured by enzyme-linked immunosorbent assay in milk before and after infusion with lipopolysaccharide or physiological saline solution. **(A)** CCL2 concentration measured in milk (*n* = 6–8). **(B)** G-CSF concentration measured in milk (*n* = 4). **(C)** CXCL1 concentration measured in milk (*n* = 4). Results are given as mean (±SD). *p < 0.05; **p < 0.01; ***p < 0.001; ****p < 0.0001 (statistical analysis: multiple *t*-test).

In a previous study, we demonstrated that infection of mice with highly virulent *E. coli* that were clinical isolates from bovine mastitis cases caused induction of a distinct profile of cytokines/chemokines, differing markedly from the corresponding profile induced by less virulent bacterial strains. In particular, highly virulent mastitis pathogens caused a profound induction of the chemokines CXCL1 and CCL2 as well as the neutrophil growth factor G-CSF. To investigate whether these compounds also are induced in the experimental bovine mastitis, we assayed for their levels in the milk of control- and LPS-infused cows. As seen in Figure 5A, CCL2 could not be detected in the milk of control cows. However, the LPS infusion caused a dramatic and significant increase in the levels of milk CCL2, first being observable 2 h after LPS infusion and persisting for at least 24 h (Figure 5A). G-CSF and CXCL1 were also detectable in milk from control cows (Figures 5B,C). However, neither of these compounds was significantly elevated after LPS infusion (Figures 5B,C).

### Intramammary LPS Infusion Does Not Cause Elevation of Histamine Levels

Previous studies have implicated mast cells in the host response against bacterial infection (36). When mast cells are activated they typically respond by degranulation, which causes the release of numerous preformed inflammatory compounds, including histamine, proteases, and proteoglycans (37). To address the potential contribution of mast cells in experimental bovine mastitis, we measured the levels of histamine in milk and plasma after intramammary LPS infusion. However, although there was a slight trend of increased histamine levels in milk and plasma after LPS infusion, this did not reach statistical significance (Figure S1 in Supplementary Material).

### Intramammary LPS Infusion Causes Changes in the Plasma Metabolome

**Figure 6:**
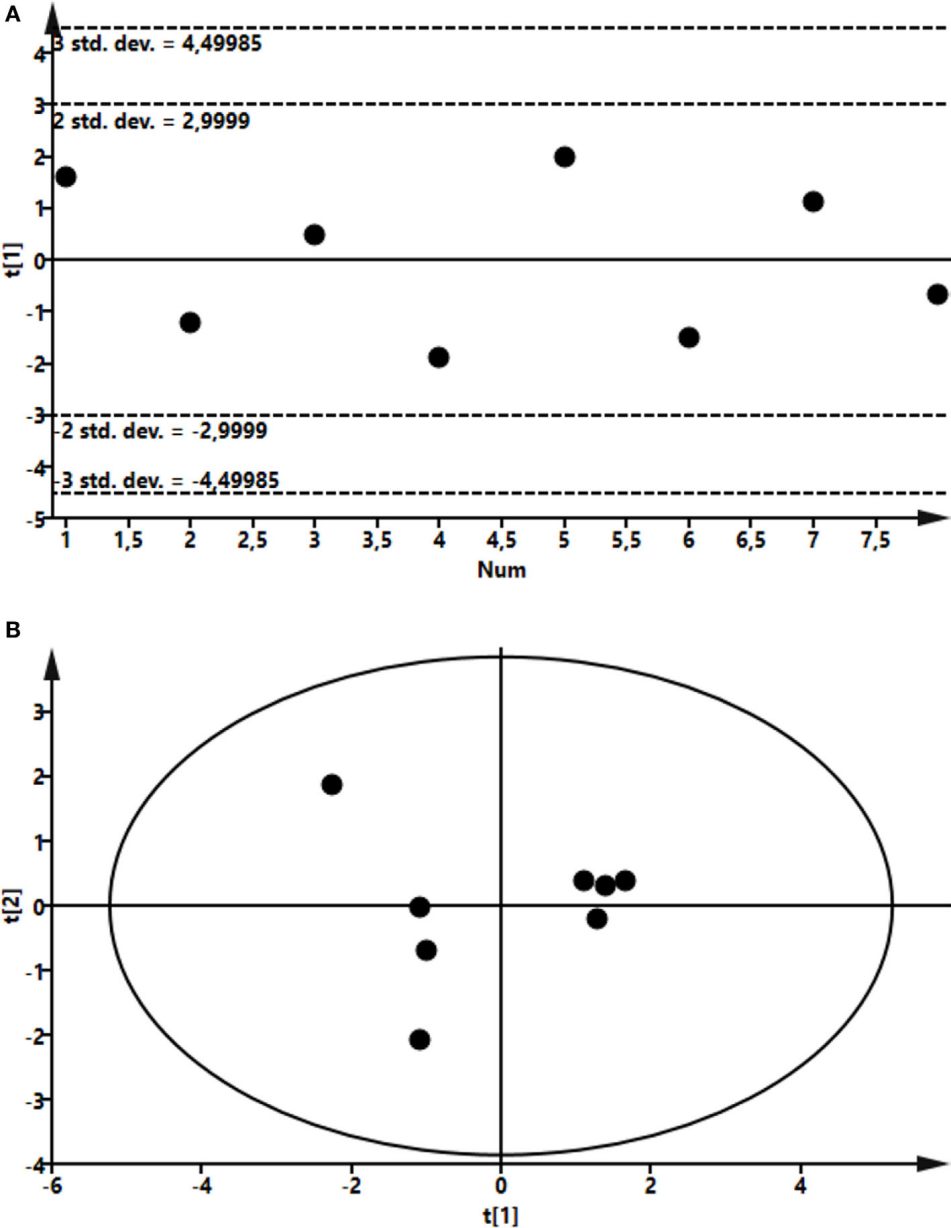
Score plot of the partial least-squares analysis (PLS) model fitted to compare plasma metabolic profile between lipopolysaccharide (LPS) and control group. Plasma was recovered from LPS-infused and control animals followed by metabolomics analysis. **(A)** Profile at 4 h. Model parameter for one component fitted were as follow: R^2^Y = 0.86; Q^2^ = 0.74; cross-validated ANOVA, *p* = 0.03. Below control, above LPS samples. **(B)** Profile at 24 h. Model parameter for three components fitted were as follow: R^2^Y = 0.98; Q^2^ = 0.93; cross-validated ANOVA, *p* = 0.08. Left control, right LPS samples.

To assess the effects of intramammary LPS infusion on metabolites in the plasma, a targeted metabolomics approach was employed. A PCA showed that the metabolome in plasma taken from control and LPS-infused animals differed profoundly, starting from 4 h after LPS infusion and persisting at 24 h (Figure 6). In total, 48 metabolites could be identified by the NMR-based metabolomics approach (Table S2 in Supplementary Material). Multivariate models were used to compare the concentrations of these 48 metabolites in plasma from LPS-infused and control animals at each time point. No outlier was indicated in the PCA model. Two partial least-squares-discriminant analysis (PLS-DA), models fitted for the 4 h (1 component; R^2^Y = 0.86; Q^2^ = 0.74) and 24 h time points (3 components; R^2^Y = 0.98; Q^2^ = 0.93) were found reliable based on their R^2^Y and Q^2^ values showing a metabolic difference between plasma from LPS-infused vs. control animals at these time points. 3-hydroxybutyrate, acetate, and lactose were found to be different in plasma from LPS-infused vs. control animals at 4 h after LPS infusion based on their VIP > 1. 3-hydroxybutyrate, citrate, and acetate were found different in plasma from LPS-infused vs. control animals at 24 h based on their VIP > 1. The metabolites, which were found different at 4 and 24 h based on their VIP > 1 were further compared between plasma from LPS-infused vs. control animals at all time-points using univariate statistics to generate a *p*-value. In addition, since changes in 3-hydroxybutarate concentrations in blood indicated a shift in ketone body metabolism in response to LPS, we further investigated acetoacetate and acetone using univariate statistics even though effects on these metabolites were not indicated through the multivariate statistics. Moreover, since changes in acetate indicated a shift in short chain fatty acids metabolism, we further investigated effects on other short chain fatty acids, i.e., butyrate and propionate using univariate statistics. Lactate was also analyzed using univariate statistics.

**Figure 7:**
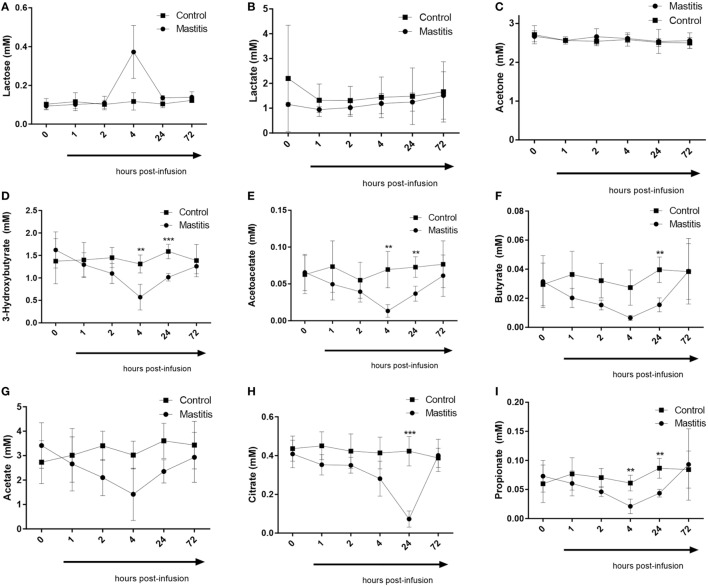
Metabolomics profile in plasma before and after infusion with lipopolysaccharide or physiological saline solution. **(A)** Lactose (*n* = 4). **(B)** Lactate (*n* = 4). **(C)** Acetone (*n* = 4). **(D)** 3-Hydroxybutyrate (*n* = 4). **(E)** Acetoacetate (*n* = 4). **(F)** Butyrate (*n* = 4). **(G)** Acetate (*n* = 4). **(H)** Citrate (*n* = 4). **(I)** Propionate (*n* = 4). Results are given as mean (±SD). *p < 0.05; **p < 0.01; ***p < 0.001; ****p < 0.0001 (statistical analysis: multiple *t*-test).

Four hours after LPS infusion, a profound increase in the plasma levels of lactose was seen. Increased levels of plasma lactose were seen in all cows of the LPS-infused vs. control cows. However, due to a large variation in the amplitude of the lactose increase, the elevation of plasma lactose did not reach statistical significance (Figure 7). We also noted that the plasma levels of the ketone bodies, 3-hydroxybutyrate, and acetoacetate, were significantly decreased after LPS infusion. A maximal decrease in the levels of these metabolites was seen 4 h after LPS infusion, but significant decreases where also seen up to 24 h (Figure 7). At 72 h after LPS infusion, the levels of ketone bodies had returned to baseline values. In contrast, the levels of acetone, i.e., the third ketone body, were not altered by intramammary LPS infusion (Figure 7). Intramammary LPS infusion was also accompanied by a substantial reduction in the levels of short-chain fatty acids: butyrate, propionate, and acetate. The reduction in these was maximal at 4 h after LPS infusion and was sustained up to 24 h. At 72 h, the levels of all short-chain fatty acids had returned to the baseline (pre-infusion) values. The reduction in both butyrate and propionate was statistically significant, whereas the reduction in acetate did not reach statistical significance (Figure 7). The largest metabolic effect of the intramammary LPS infusion was seen for citrate, which was reduced by ~80% 24 h after LPS infusion (Figure 7).

### The Milk Metabolome Is Affected by Intramammary LPS Infusion

To compare the profile of metabolites in milk in LPS-infused vs. control cows, we performed an untargeted metabolomics approach. Multivariate models were used to compare the bucketed data from the NMR spectra of milk samples from LPS-infused vs. control animals at each time point. No outlier was indicated in the PCA model. Two PLS-DA models fitted for the 24 h (2 components; R^2^Y = 0.94; Q^2^ = 0.80) and 72 h time points (2 components; R^2^Y = 0.96; Q^2^ = 0.88) were found reliable based on their R^2^Y and Q^2^ values showing a metabolic difference in milk from LPS-infused vs. control animals at these time points. NMR signals corresponding to lactose and lactate at 24 h and NMR signals corresponding to lactose at 72 h were found different between LPS-infused and control animals based on VIP > 1. For further univariate statistical investigation, the intensity of signal at 3.945 and 1.355 ppm were used for lactose and lactate, respectively.

**Figure 8:**
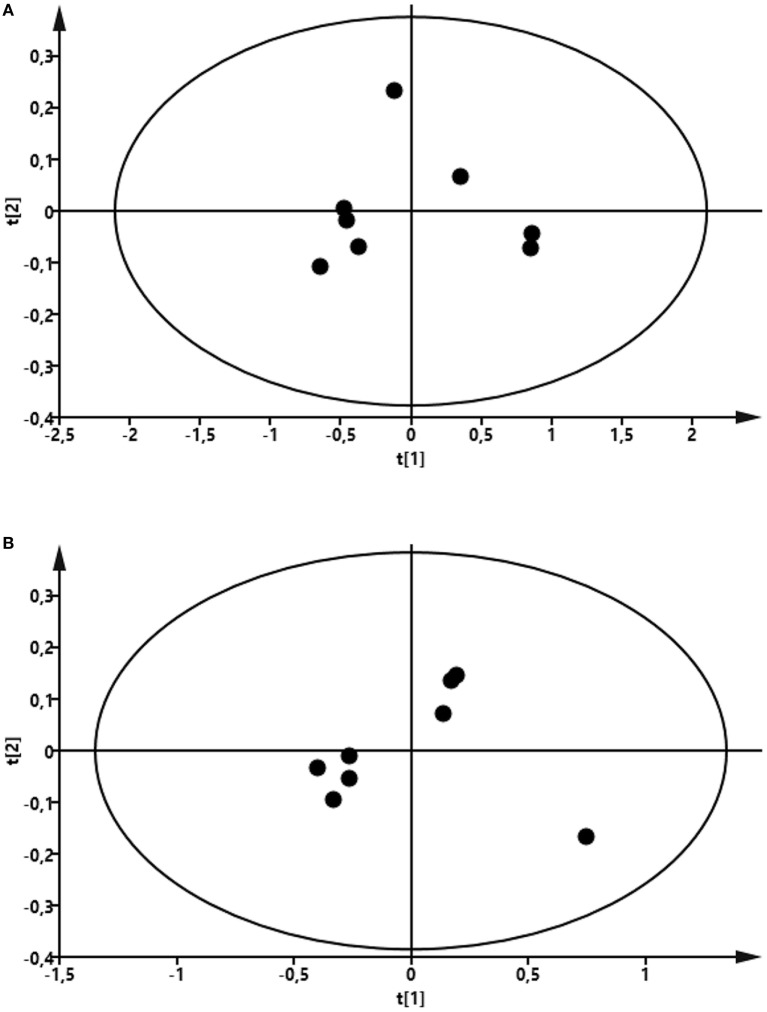
Score plot of the partial least-squares analysis (PLS) model fitted to compare milk metabolic profile between lipopolysaccharide (LPS) and control group. Milk was taken from LPS-infused and control animals followed by metabolomics analysis. **(A)** Profile at 24 h. Model parameter for two components fitted were as follow: R^2^Y = 0.94; Q^2^ = 0.80; cross-validated ANOVA, *p* = 0.06. Left control, right LPS samples. **(B)** Profile at 72 h. Model parameter for two components fitted were as follow: R^2^Y = 0.96; *Q*^2^ = 0.88; cross-validated ANOVA, *p* = 0.03. Left control, right LPS samples.

**Figure 9:**
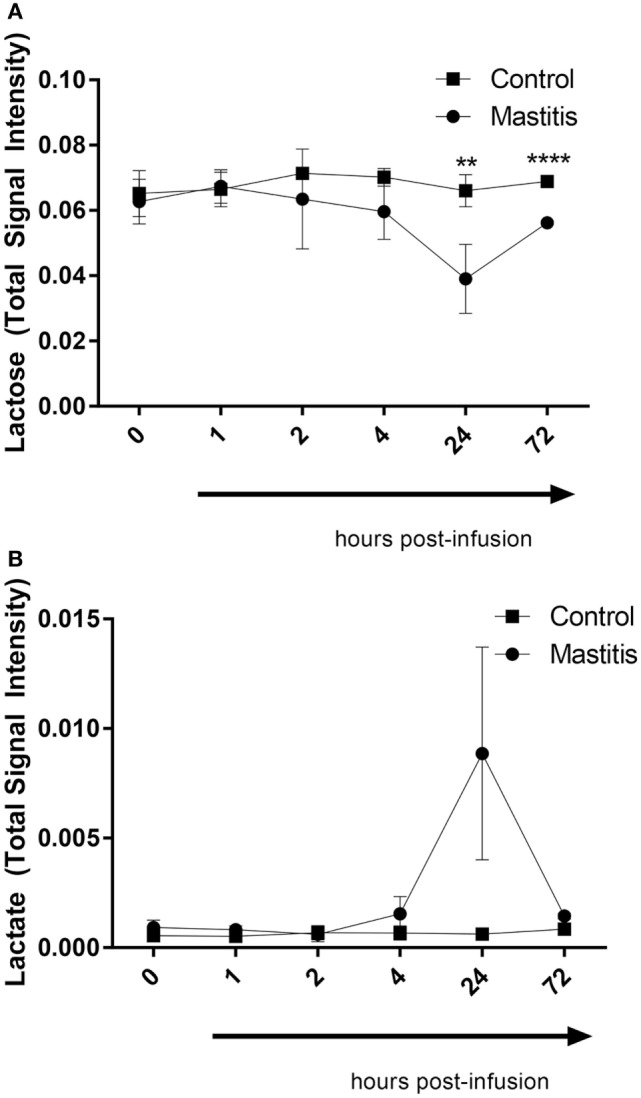
Lactose and lactate levels in milk before and after infusion with lipopolysaccharide or physiological saline solution. **(A)** Lactose (*n* = 3–4). **(B)** Lactate (*n* = 4). Results are given as mean (±SD). *p < 0.05; **p < 0.01; ***p < 0.001; ****p < 0.0001 (*n* = 4) (statistical analysis: multiple *t*-test).

As seen in Figure 8, the PCA revealed that LPS infusion caused a major shift in the milk metabolome. As seen in Figure 9, the intramammary LPS infusion caused a significant reduction in the levels of milk lactose and a strong trend of increased lactate content. Increased lactate levels were seen in the milk of all LPS-infused vs. control cows (Figure 9). However, due to the large variation in the amplitude of increase, the difference between the samples from LPS-infused vs. control cows did not reach statistical significance.

## Discussion

In this study, we performed a kinetic assessment of multiple parameters after intramammary infusion of LPS. The investigated parameters included concentrations of inflammatory mediators in milk and plasma, as well as changes in the concentrations of milk and plasma metabolites. To our knowledge, this comprehensive approach has not been used before. Instead, previous studies have primarily focused on monitoring individual parameters.

In this work, we observed a rapid onset of clinical signs of mastitis after LPS infusion, including a dramatic rise in rectal temperature, local pain perception, swelling of udder, and heat perception. For all of these clinical parameters, the peak was seen after 2 h, followed by a return to baseline conditions within 24 h, in agreement with previous studies of how LPS induces clinical signs of mastitis (38–40). The LPS infusion also produced profound milk changes, in agreement with previous findings in both clinical and LPS-induced mastitis (1, 41). Interestingly, the effects on milk, i.e., visible milk changes and SCC, were observed with a distinct delay as compared with the clinical signs. Whereas the general clinical parameters (heat, pain, swelling, rectal temperature) peaked at 2–4 h followed by a rapid decline, the changes in milk parameters were first observed at 4 h after LPS infusion, peaked at 24 h, and persisted until at least 72 h. There was also a marked delay in the metabolic changes in milk vs. those seen in the plasma. Hence, the systemic effects and the local effects in the udder tissue preceded the development of the clinical parameters in the milk.

When assessing the cytokine response following LPS infusion, the kinetics were relatively similar to the development of systemic clinical parameters and local clinical signs in the udder. In plasma, a profound increase in IL-6 was seen at 4 h post-infusion, i.e., at the time point where the maximal clinical score was observed, and there was also a trend of an increase in plasma TNF-α at this time point. The intramammary LPS infusion also caused a profound increase in the levels of IL-6 and TNF-α in the milk. However, the increase in these cytokines was markedly delayed in comparison with their increase in plasma. Clearly, this is in agreement with a later onset of inflammatory signs in the milk as compared with the systemic effects of intramammary LPS infusion. These findings are in line with several previous studies showing that IL-6 and TNF-α are elevated in mastitis (42–45). With regard to TNF-α and its association with bovine mastitis, there are partly inconsistent reports. In agreement with our findings, one study found an increase of TNF-α in the milk but not in blood (46) whereas another study showed elevated levels of TNF-α in the blood (47). One potential explanation for these inconsistencies could be that the different studies utilized different LPS doses: 100 µg in the studies where TNF-α was not found to be elevated in plasma [the present study and (46)] and 200 µg in the study where an increase in plasma TNF-α was seen (47).

In milk, we also observed a large increase in the levels of CCL2. CCL2 has previously been implicated in mastitis through several *in vitro* studies showing that CCL2 can be induced in bovine mammary tissue and epithelial cells by LPS, lipoteichoic acid, or by formalin-killed *Staphylococcus aureus* (48–51). However, to our knowledge, our study is the first to show that CCL2 is upregulated *in vivo* during acute mastitis. Interestingly, the LPS-induced induction of CCL2 preceded the increase in SCC, a finding that is clearly compatible for a role of CCL2 in recruiting inflammatory cells to the udder. In contrast, the onset of visible milk changes occurred with similar kinetics as for CCL2 induction, arguing that the induction of this chemokine is not causative in producing the visible milk changes. Based on these findings, CCL2 may thus be considered as a potential inflammatory biomarker for acute mastitis. However, further assessments of CCL2 levels in clinical and subclinical mastitis would be warranted for this purpose.

CCL2 is a powerful monocyte chemoattractant (52) and macrophages are considered to be the predominant immune cell type in milk alongside neutrophils. Macrophages are present in the milk of healthy udders and monocytes are recruited into the udder following the primary neutrophil response to bacterial insult. Monocytes differentiate into macrophages and participate in the clearance of neutrophils during the resolution of the inflammation (2). The increase of CCL2 in the mastitic milk suggests the possibility that it could be involved in the recruitment of monocytes to the mammary gland.

Mast cells have been implicated in the host response to bacterial insult (36) and are present in the bovine udder where they have been considered to be the major source of histamine (53, 54). Moreover, it has been reported that histamine levels are elevated in the milk of cows afflicted by clinical mastitis (55). Based on these notions, we assessed the involvement of mast cells in LPS-induced acute mastitis by measuring the levels of histamine in plasma and milk. However, no significant elevation in the histamine levels were seen at either of these sites, arguing against a major contribution of mast cell activation in the response toward LPS. Alternatively, we cannot exclude that mast cells may actually contribute to the mastitis although not undergoing degranulation to a large extent. For example, LPS-activated mast cells are known to release IL-6 and TNF-α without concomitant degranulation (56), and we can thereby not rule out that mast cells contribute to the elevation in the levels of these cytokines following LPS infusion.

A major focus of this study was to follow the effects of intramammary LPS infusion on the metabolome of the milk and plasma. Interestingly, changes in the plasma metabolome occurred with delayed kinetics in comparison with the onset of clinical signs and plasma cytokine induction, with maximal effects on most of the affected metabolites seen at 4 h after LPS infusion. This suggests that the plasma metabolome changes occur as an event secondary to the observed clinical signs and plasma cytokine induction. One of the findings from these analyses was an elevation in the levels of plasma lactose after LPS infusion. This is in agreement with previous studies showing that disruption of tight junctions in mammary epithelial cells occurs during mastitis, causing an influx of lactose over the blood:milk barrier leading to increased levels of lactose in plasma (57). Notably, the peak in plasma lactose was seen 4 h after LPS infusion and coincided with the peak of IL-6 induction in plasma, arguing that the increase in plasma lactose is linked to the appearance of systemic inflammatory signs. We also noted a corresponding decrease in the levels of lactose in the milk, most likely being a consequence of increased leakage from the milk to the general circulation.

Another interesting finding was that the intramammary infusion of LPS caused a marked drop in the levels of short-chain fatty acids (in particular propionate and butyrate) in plasma. Short-chain fatty acids are known to be produced in large quantities by the rumen microbiota. Our findings thus introduce the possibility that the acute mastitis induced by LPS infusion affects the rumen microbiota populations. We cannot at present with certainty explain the mechanism behind these findings. However, one possible scenario is that the acute mastitis causes a reduced output of short-chain fatty acids from the gut microbiota, although the mechanism behind such a scenario will warrant a further investigation.

We also noted that the intramammary infusion of LPS caused a decrease in the levels of ketone bodies in plasma (3-hydroxubutyrate and acetoacetate). Bovine plasma concentrations of 3-hydroxybutyrate are under normal conditions to a large extent dependent of rumen butyrate production as butyrate is converted to 3-hydroxybutyrate while passing over the rumen wall. The decline in 3-hydroxybutyrate could thus partly be explained by a decreased butyrate production from the rumen microbiota (see above).

Among the effects seen on the metabolome, the most dramatic one was a decrease (~80%) in the levels of plasma citrate. Citrate is produced by the condensation of acetyl-coenzyme A and oxaloacetate in the first step of the citric acid cycle. However, citrate is also (after transport from the mitochondria to the cytoplasm) the precursor for fatty acid synthesis. Potentially, a decline in the levels of citrate could thus reflect a lower rate of fatty acid synthesis. The liver and the udder are primary sites for fatty acid synthesis, and it is hence possible that the decreased citrate levels can be due to hampered liver or udder function as a consequence of the inflammatory conditions induced by LPS infusion. In line with the findings presented here, it was shown in a previous study that clinical mastitis was associated with a decrease of citrate in the milk (19), and decreased citrate levels have also been observed in subclinical mastitis (58). Our findings are thus consistent with these latter studies and, based on these studies together, citrate can be considered as a potential biomarker that can be useful in the monitoring of mastitis.

In conclusion, this study provides a kinetic overview of the inflammatory and metabolic changes that are invoked during experimentally induced bovine mastitis. Our data show that intramammary LPS infusion rapidly causes clinical signs of acute mastitis, which was paralleled in time with the clinical signs of acute mastitis, whereas changes in the plasma metabolome occurred with a delay. Intramammary LPS infusion also caused multiple effects on the milk, including increased SCC, cytokine output, and effects on the metabolome. However, all of these changes in milk parameters occurred with delay in comparison with corresponding parameters in plasma.

## Ethics Statement

This study was carried out in accordance with the recommendations of the local ethics committee. The protocol was approved by the Uppsala djurförsöksetiska nämnd (ethical permit C22/15).

## Author Contributions

C-FJ planned the *in vivo* work and monitored temperature, processed samples, performed analyses, interpreted data, and wrote the manuscript; JD planned the experimental *in vivo* work, performed milk sampling, blood sampling, clinical scoring, and the intramammary infusions; A-MG participated in sampling, sample processing, and data recording; IW performed analyses and interpreted data; AM performed metabolomic analyses, interpreted data and contributed to the writing of the manuscript; KÖ participated in the planning of the study, took part during the infusions, and contributed to the writing of the manuscript; GP conceived of and planned the study, interpreted data, and wrote the manuscript.

## Conflict of Interest Statement

The authors declare that the research was conducted in the absence of any commercial or financial relationships that could be construed as a potential conflict of interest.

## Supplementary Material

Figure S1 Concentration of histamine measured by enzyme-linked immunosorbent assay in milk and plasma before and after infusion with lipopolysaccharide or physiological saline solution. **(A)** Histamine concentration measured in milk (*n* = 8). **(B)** Histamine concentration measured in plasma (*n* = 6–8). Results are given as mean (±SD). **p* < 0.05; ***p* < 0.01; ****p* < 0.001; *****p* < 0.0001. Statistical analysis: multiple t-test.

## Abbreviations

LPS, lipopolysaccharide; NMR, nuclear magnetic resonance; SCC, somatic cell count; IMI, intramammary infection; ELISA, enzyme-linked immunosorbent assay; BSA, bovine serum albumin.
