# Supplementary figures and images for: The Effect of Lipopolysaccharide-Induced Experimental Bovine Mastitis on Clinical Parameters, Inflammatory Markers, and the Metabolome: A Kinetic Approach
Source: Front Immunol. 2018 Jun 25;9:1487. doi: 10.3389/fimmu.2018.01487 (PMC6026673; doi:10.3389/fimmu.2018.01487)

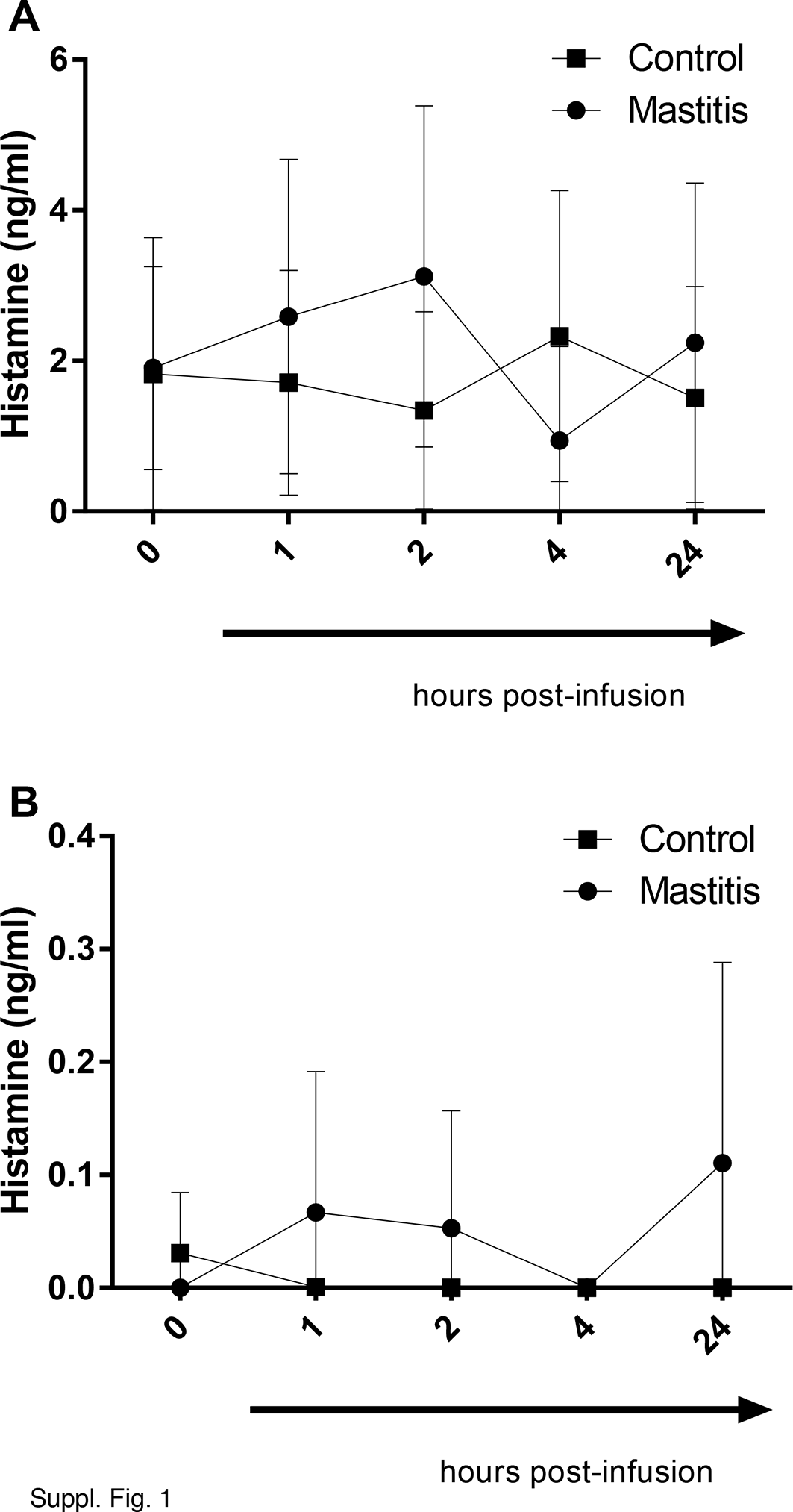

Supplement: Figure S1 — Concentration of histamine measured by enzyme-linked immunosorbent assay in milk and plasma before and after infusion with lipopolysaccharide or physiological saline solution. (A) Histamine concentration measured in milk (n = 8). (B) Histamine concentration measured in plasma (n = 6–8). Results are given as mean (±SD). *p < 0.05; **p < 0.01; ***p < 0.001; ****p < 0.0001. Statistical analysis: multiple t-test. [file Supplementary_Figure_S1.TIF]
