# Supplementary material for: The Effect of Lipopolysaccharide-Induced Experimental Bovine Mastitis on Clinical Parameters, Inflammatory Markers, and the Metabolome: A Kinetic Approach
Source: Front Immunol. 2018 Jun 25;9:1487. doi: 10.3389/fimmu.2018.01487 (PMC6026673; doi:10.3389/fimmu.2018.01487)
Supplement: Supplementary file 2 [file Table_1.DOCX]

**Supplementary Table 1. Clinical scoring for signs of udder inflammation heat, redness, swelling, pain and milk changes.** The scoring was based on comparing an infused quarter with a health quarter on the same animal (heat, redness, swelling) or by comparing the response of an animal to manual milking pre- and post-infusion (pain). Scoring milk changes was based on the colour and the presence of clots.

|  | **0** | **1** | **2** | **3** |
| --- | --- | --- | --- | --- |
| **Heat** | No change | Low perceptible temperature increase | Moderate perceptible temperature increase | High perceptible temperature increase |
| **Pain** | No change | Moderately irritated | Moderately irritated  Kicks | Highly irritated  Kicks |
| **Redness** | No change | Light redness | Moderate redness | Intense redness |
| **Swelling** | No change | Perceptible hardening of the gland tissue | Moderate hardening of the gland tissue | Severe hardening of the gland tissue |
| **Milk Changes** | No change | Yellow | Yellow  Clots | Intensely yellow  Clots |
