# Supplementary material for: The Effect of Lipopolysaccharide-Induced Experimental Bovine Mastitis on Clinical Parameters, Inflammatory Markers, and the Metabolome: A Kinetic Approach
Source: Front Immunol. 2018 Jun 25;9:1487. doi: 10.3389/fimmu.2018.01487 (PMC6026673; doi:10.3389/fimmu.2018.01487)
Supplement: Supplementary file 3 [file Table_2.docx]

**Supplementary Table 2**. Metabolites identified in bovine plasma.

| **#** | **Metabolite** |
| --- | --- |
| **1** | 2-Aminobutyrate |
| **2** | 2-Hydroxy-3-methylvalerate |
| **3** | 2-Hydroxybutyrate |
| **4** | 2-Hydroxyvalerate |
| **5** | 2-Oxovalerate |
| **6** | 3-Hydroxybutyrate |
| **7** | Acetate |
| **8** | Acetoacetate |
| **9** | Acetone |
| **10** | Alanine |
| **11** | Arginine |
| **12** | Asparagine |
| **13** | Betaine |
| **14** | Butyrate |
| **15** | Carnosine |
| **16** | Choline |
| **17** | Citrate |
| **18** | Creatine |
| **19** | Creatinine |
| **20** | Dimethyl sulfone |
| **21** | Ethanol |
| **22** | Formate |
| **23** | Glucose |
| **24** | Glutamate |
| **25** | Glutamine |
| **26** | Glycerol |
| **27** | Glycine |
| **28** | Hippurate |
| **29** | Histidine |
| **30** | Isoleucine |
| **31** | Lactate |
| **32** | Lactose |
| **33** | Leucine |
| **34** | Lysine |
| **35** | Methionine |
| **36** | Phenylalanine |
| **37** | Proline |
| **38** | Propionate |
| **39** | Propylene glycol |
| **40** | Pyruvate |
| **41** | Serine |
| **42** | Taurine |
| **43** | Threonine |
| **44** | Trimethylamine N-oxide |
| **45** | Tyrosine |
| **46** | Valerate |
| **47** | Valine |
| **48** | myo-Inositol |
